# Comparison of manual and semi-automated algorithm for measuring architectural features during different isometric knee extension intensities: a reliability and comparative study in novice raters

**DOI:** 10.3389/fresc.2025.1539804

**Published:** 2025-04-03

**Authors:** Micheal J. Luera, JoCarol E. Shields, Emma Bozarth, Rob J. MacLennan, Natalie P. Walker, Jesus A. Hernandez-Sarabia, Carlos A. Estrada, Jason M. DeFreitas, Scott K. Crawford

**Affiliations:** ^1^ Department of Neuroscience, Tarleton State University, Stephenville, TX, United States; ^2^ Department of Health and Human Performance, Tarleton State University, Stephenville, TX, United States; ^3^ Neural Health Research Laboratory, Syracuse University, Syracuse, NY, United States; ^4^ Malcom Randall VA Medical Center, Gainesville, FL, United States; ^5^ Department of Neurology, University of Florida, Gainesville, FL, United States; ^6^ Department of Exercise Science, Aurora University, Aurora, IL, United States; ^7^ Department of Kinesiology, University of Wisconsin-Madison, Madison, WI, United States; ^8^ Department of Orthopedics and Rehabilitation, University of Wisconsin-Madison, Madison, WI, United States; ^9^ Department of Biomedical Engineering, University of Wisconsin-Madison, Madison, WI, United States

**Keywords:** ultrasound, muscle, fascicle length, pennation angle, reliability

## Abstract

**Introduction:**

Ultrasound is a cost-effective and reliable method to determine skeletal muscle architecture. However, manual analysis of fascicle length (FL) and pennation angle (PA) can be arduous and subjective among raters, particularly among novice raters. Alternatives to manual processing have been proposed that expedite the evaluation of muscle architecture and afford more consistency. While using algorithms has provided dependable results of muscle architecture, it has often focused on variables of passive range of motion and submaximal contractions. To fully understand the impact of muscle architecture using semi-automated analysis, an investigation of a broad range of contraction intensities is needed. The purpose of this study was to develop and determine the intra-rater and inter-rater reliability of a custom, semi-automated algorithm to extract measures of muscle thickness, pennation angle, and fascicle length, and second to compare the semi-automated measures to measures extracted manually from the same novice raters while accounting for differences between contraction intensities.

**Methods:**

Fifteen resistance-trained individuals (male: *n* = 6, female: *n* = 9) completed this study. Images were collected during four contraction intensities relative to maximal voluntary isometric contractions (MVIC) (at rest, 30%, 70%, and MVIC) and analyzed by three novice raters to compare the semi-automated algorithm and manual measurement in the vastus lateralis.

**Results:**

Intra-rater reliability for manual measures was poor for FL (ICCs: 0–0.30), poor to good for PA (ICCs: 0.46–0.77), and moderate to good for muscle thickness (MT) (ICCs: 0.55–0.84). For the semi-automated algorithm, the intra-rater reliability was good to excellent for FL (range: 0.90–0.99), PA (range: 0.88–0.99), and MT (range: 0.996–0.999) across all contraction intensities.

**Discussion:**

The findings of this study suggest that the reliability of manual measurement is lower when novice raters perform image analyses compared to the semi-automated method. Therefore, careful consideration and training should be provided when considering manual assessment of muscle architecture values, and standardized identification methods and features in algorithm development may be a better method for reproducibility.

## Introduction

Muscle structure is a key determinant in the functional capacity of the muscle to generate and transmit force. Morphological features such as muscle thickness (MT), anatomical cross-sectional area, and volume are often assessed using medical imaging techniques such as magnetic resonance imaging (MRI) or ultrasound (1–3). Architectural features, such as pennation angle (PA) and fascicle length (FL), are related to the physiological cross sectional area which is directly proportional to maximal tetanic tension (4, 5). Considering the relationship between muscle architecture, force generation, and excursion, morphological and architectural features are often measured to characterize adaptations in response to strength training, injury, or aging (6–11).

Both MRI and ultrasound imaging provide insight into morphological and architectural features in a non-invasive manner (12). MRI is the gold standard for measures of muscle thickness, anatomical cross-sectional area, and volume, especially for deeper muscles, but macro-morphological measures made with ultrasound imaging, such as muscle thickness, have also been shown to be reliable compared to MRI (13, 14). Due to the real-time imaging capabilities, portability, ease of use, and cost compared to MRI, ultrasound has significant advantages which makes this modality more feasible to use in outpatient clinical settings and when combined with other equipment to assess muscle function (e.g., isokinetic dynamometers).

Architectural measures of FL and PA are typically measured using ultrasound, even in studies using MRI to characterize physiological cross-sectional area, though MRI diffusion tensor imaging sequences have also been used to determine FL (1, 4, 15–20). Although the reliability of extracting macromorphological and architectural features is high when using ultrasound, measuring FL and PA are performed manually (21). This is tedious and time consuming, particularly in studies with large sample sizes or with repeated measures, and usually only include a few representative fascicles for the PA measures, which introduces some level of subjectivity. Additionally, ultrasound measures are user-dependent, which indicates a high level of training is often necessary and measures from novice raters are subject to high variability (22).

Some groups have developed different algorithms or quantitative image analysis techniques to characterize muscle architectural features in different muscles (23–27). However, most have focused on the reliability of these measures either between days or relative to other imaging techniques (23, 28–31). Additionally, these algorithms have been tested in passive range of motion and submaximal (∼10%) contractions of the vastus lateralis muscle, but have not assessed reliability across a range of contraction intensities (23, 26, 32). Typical quantitative methods will often measure pennation angle relative to the horizontal, which does not account for any angulation of the deep aponeurosis which could be problematic in contracted conditions (33). This is particularly noteworthy considering the changes in pennation angle with contraction intensity (33–36). Considering manual analysis is the typical method for measuring PA and FL, the purpose of this study was two-fold: (1) to develop and determine the intra-rater and inter-rater reliability of a custom, semi-automated algorithm to extract measures of muscle thickness, pennation angle, and fascicle length, and (2) to compare the semi-automated measures to measures extracted manually from the same novice raters while accounting for differences between contraction intensities.

## Materials and methods

### Human participants

Fifteen resistance-trained men (*n* = 6, mean ± SD age = 24 ± 3 yrs, mass = 88.53 ± 11.58 kg, height = 175.48 ± 5.57 cm) and women (*n* = 9, 22 ± 2 yrs, 65.26 ± 2.82 kg, 167.06 ± 4.860 cm) completed this study. All subjects visited the laboratory on two separate occasions, each separated by 48–72 h. The initial visit involved a physical activity readiness questionnaire (PAR-Q), informed consent, and familiarization of isometric strength testing procedures. Inclusion criteria required no previous or current neurological and/or musculoskeletal injuries of the lower extremities and the ability to barbell back squat at least 1.5 times their body weight. This investigation was approved by the Oklahoma State University Intuitional Review Board for the protection of human subjects (IRB Approval #ED1783).

### Isometric testing

Each visit began with a standardized warm-up involving 5 min on a cycle ergometer, 10 self-paced body-weight squats, and 3 moderate intensity isometric knee extensions (KE) at 30, 50, and 75% of their perceived maximum. For all isometric KE contractions, force was recorded from the right leg using an S-beam load cell (Model SSM-AJ-500; Interface, Scottsdale, AZ, USA) attached to a cuff around the ankle. Subjects were seated in an upright position with their knee joint angles positioned at 110° of flexion (0° = full extension).

Following warm-up, subjects completed a 5 s maximal voluntary isometric contraction (MVIC). Peak force production was taken during a 0.5 s epoch and used to project target force trajectories for subsequent force tracings. Submaximal ramp contractions were then performed at 30% and 70% of relative MVIC force. Real-time force feedback was provided during the submaximal contraction, which allowed the participants to accurately produce a trapezoidal force trajectory that included a linear increasing rate of 10%/s force, a steady force hold for 10 s at the targeted 30% and 70% MVIC, and finally a −10%/s linearly decreasing segment back to baseline. Each contraction was separated by two minutes of rest. Ultrasound images were taken during the peak force hold for the MVC and during the plateau of the force hold in submaximal tracings.

### Ultrasonography

During the testing visit, PA of the vastus lateralis was assessed using brightness mode (B-mode) ultrasound imaging (General Electric LOGIQ S8, Wauwatosa WI, USA) using a multifrequency linear-array probe (Model ML6-15-D MHz, 50 mm field of view). All images were taken by a single researcher (MJL) at rest and during each of the contractions (i.e., 30%, 70%, MVC) along the sagittal plane at 50% of the distance from the anterior superior iliac spine to the lateral side of the patella. A high-density foam pad was used to secure the probe to the leg of the subject to minimize probe movement during contractions. Each image was collected by the same investigator, using a generous amount of water-soluble transmission gel, and adjusting for consistent minimal pressure during all applications. Image gain was set at 50 decibels (dB) with the dynamic range set at 72 dB. Depending on the subcutaneous tissue thickness of the thigh, the image depth was set at either 5 or 6 cm.

### Algorithm development and image analysis

**Figure 1:**
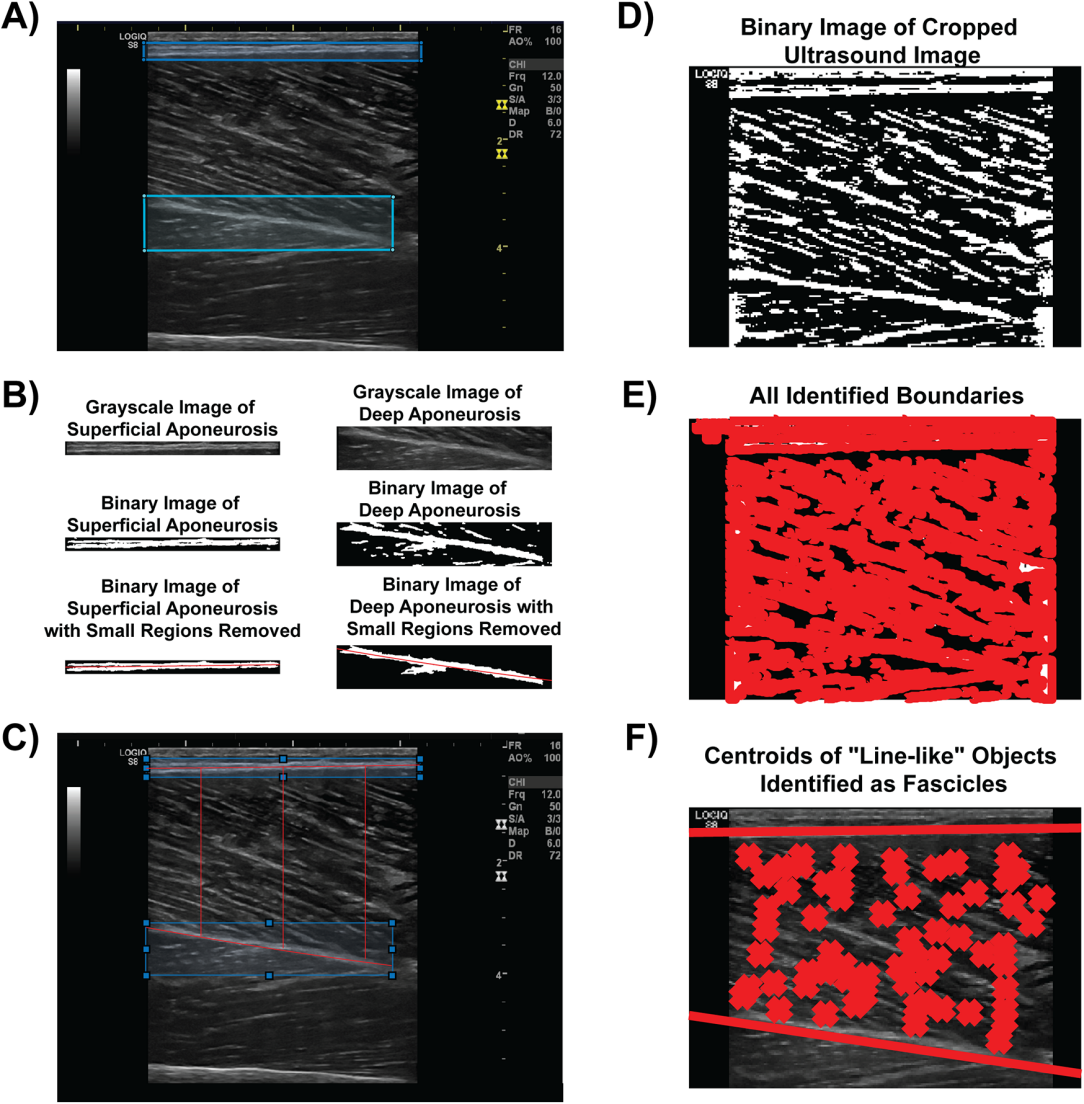
Semi-automated algorithm analysis procedure. **(A)** The user identifies and manually circumscribes the superficial (blue) and deep (cyan) aponeuroses from the B-mode image. **(B)** The aponeuroses are isolated, converted to a binary image, and have small regions removed to facilitate polynomial best fit (both linear and second order) for architectural feature and muscle thickness measures. **(C)** Muscle thickness measures visualized at 20%, 50% and 80% widths of the transducer aperture. **(D)** The B-mode image is converted to a binary image for boundary identification. **(E)** An example of every boundary detected within the converted binary image. **(F)** Linear fits of the superficial and deep aponeuroses are shown with line-like features (i.e., fascicles) between the aponeuroses marked with an “X”. Architectural measures were then determined from these fascicles.

The algorithm was developed using MATLAB software (The Mathworks, Natick MA). First, the user manually identified the parent region of interest (ROI) from which architectural measures and muscle thickness were calculated. Second, the user identified and separately sectioned the superficial and deep aponeuroses of the vastus lateralis muscle in each condition (Figure 1A). Each aponeurosis was then converted into a binary image using the *imbinarize* and *bwmorph* functions from the Image Processing Toolbox (Figure 1B). From the binary images, both a first- and second-order polynomial fit was created using the midpoint of the aponeurosis thickness. Small regions that were discontinuous with the aponeuroses were excluded from the binary image used for both of these polynomial fits (Figure 1B). The first-order polynomial was used to determine pennation angle and fascicle length (see description below) while the second-order polynomial fit was used to account for any curvature in the muscle when calculating muscle thickness, particularly during the %MVIC conditions. Using a custom script that calculated the calibration factor (which corresponded to approximately 10.2 pixels/mm for all images in the current study), the muscle thickness was calculated between the second-order polynomial fits between the superficial and deep aponeurosis binary images at the 20%, 50% and 80% widths of the transducer aperture (Figure 1C).

The original parent ROI was converted into a binary image using the *imbinarize* function with “adaptive” method threshold in order to calculate PA and FL (Figure 1D). All hyperechoic regions that were above the threshold value for the binary image were traced using the *bwboundaries* function (Figure 1E). Specific region properties, including the area, orientation, centroid location, eccentricity, and major axis length, of each boundary of the hyperechoic regions were extracted. The orientation property from the *bwboundaries* function determines the angle of the region with respect to the image horizontal. Therefore, the orientations of the linear polynomial fit for the deep and superficial aponeuroses, which were determined from the slopes of each line, accounted for any angulation in the aponeuroses during the various %MVIC conditions in relation to the orientation of the fascicles.

Once fascicles were determined from the *bwboundaries* function, we only included fascicles whose hyperechoic regions had an area between 75 and 6,000 pixels, an eccentricity of at least 0.90, and the centroid location between the first order line fit of the superficial and deep aponeuroses. Each fascicle that met these initial criteria was then linearly extrapolated along the length of the muscle. If the extrapolated line intersected both the superficial and deep aponeuroses, then the pennation angle with respect to the deep aponeurosis orientation was included for analysis (Figure 1F). Determination of the intersection points was optimized using the freely-available *Intersects* function from the MATLAB file exchange (37). The fascicle length was calculated from the intersection points of the fascicle and aponeuroses as $\sqrt{{\left(x_{Deep}-x_{Superficial}\right)}^{2}+{\left(y_{Deep}-y_{Superficial}\right)}^{2}}$ and expressed in terms of cm. The muscle thickness, PA, and FLs across the entire parent ROI were recorded and exported into an Excel worksheet for subsequent analysis.

All raters were trained in the procedures for the semi-automated analysis. This training included an overview of the algorithm steps and procedures (detailed above), identification of anatomical structures (i.e., aponeuroses) within the images, ROI selection for each structure, architectural (FL and PA) and thickness calculations, and exporting the data for subsequent analysis. A single training session that included all participants and the algorithm developer (SKC) was initiated prior to image analysis and lasted approximately 30 min.

### Image analysis and intra- and inter-rater reliability procedures

Images were manually analyzed using image analysis software (ImageJ, version 1.50i) available from the National Institutes of Health (NIH, Bethesda, MD). Consistent with previous methods, three unique fascicles were identified within each image and the angle at which the fascicles inserted into the deep aponeurosis was measured as the pennation angle (28, 29). The fascicle length was determined by measuring the linear path of the same fascicles used for pennation angle measures between the superficial and deep aponeuroses after accounting for the calibration tool within ImageJ which determined the number of pixels per mm within each image. Fascicle length was then calculated as FL = MT/sin(PA). Muscle thickness was determined by the vertical distance between the superficial and deep aponeuroses.

Three separate novice raters manually measured the FL, PA, and muscle thickness from the same image of the same subject. It was deemed pertinent to have different raters who had not previously analyzed the data manually run the semi-automated analysis to maintain a comparable level of training (i.e., novice) between those who performed the manual measures. The raters were familiar with basic muscle anatomy structure. Each of the novice raters was instructed to follow the guidelines based on previous literature (38). Raters practiced the image analysis procedures in training set of images comprised of approximately 20 images. One rater (EB) was also randomly selected to repeat the manual image analysis for all images on a separate day. An additional three separate novice raters used the semi-automated algorithm for extracting FL, PA, and muscle thickness from the same images from which manual measures were derived. One rater (NW) repeated the procedures on a different day from when the initial measures were acquired. Measures derived from the manual and semi-automated algorithm analyses were exported into an Excel worksheet for subsequent analysis.

### Statistical analysis

The intraclass correlation coefficient (ICC) was calculated for both intra- and inter-rater reliability (39). A two-way mixed effects, single rater (with absolute agreement considered to be important) ICC was calculated for the intra-rater reliabilities (manual and semi-automated) for all architectural measures. A two-way random effect, multiple raters (with absolute agreement considered to be important) ICC [ICC(2,k)] was calculated for the inter-rater reliabilities (manual and semi-automated). The 95% confidence intervals for each ICC were also calculated. The ICCs were interpreted based upon previous recommendations with values <0.50 indicating poor reliability, 0.50–0.75 indicating moderate reliability, 0.75–0.90 indicating good reliability, and >0.90 indicating excellent reliability (39). Pearson's correlations were determined for both manual and semi-automated intra-rater measures. Bland-Altman statistics were also calculated using the *blandr.statistics* function from the *blandr* package to determine the bias between manual measures extracted from the novel raters and the semi-automated measurements. All statistical analyses were performed in RStudio with *a priori* significance set at *α* = 0.05.

## Results

### Manual architectural measures

The intra-rater ICC values by each contraction intensity level are presented in Table 1. The intra-rater reliability for the novice rater manual measures of FL was poor with ICCs ranging from 0–0.30 and the reliability for manual pennation angle measures was poor to good with ICCs ranging from 0.46–0.77. The reliability for muscle thickness measures was moderate to good with ICCs ranging from 0.55–0.84.

**Table 1:** Intra- and inter-rater reliability measures of fascicle length, pennation angle, and muscle thickness. Data are presented as ICC (95% confidence interval).

Contraction Intensity	Fascicle length				Pennation angle				Muscle thickness			
	Intra-rater		Inter-rater		Intra-rater		Inter-rater		Intra-rater		Inter-rater	
	Manual	Semi-automated	Manual	Semi-automated	Manual	Semi-automated	Manual	Semi-automated	Manual	Semi-automated	Manual	Semi-automated
Rest	0.25 (0, 0.67)	0.90 (0.73, 0.96)	0.75 (0.36, 0.91)	0.89 (0.72, 0.96)	0.77 (0.46, 0.92)	0.92 (0.72, 0.97)	0.85 (0.54, 0.95)	0.96 (0.88, 0.98)	0.65 (0.23, 0.86)	0.999 (0.997, 1.00)	0.92 (0.81, 0.97)	0.98 (0.94, 0.99)
30% MVIC	0 (0, 0.42)	0.95 (0.86, 0.98)	0.67 (0.21, 0.88)	0.96 (0.91, 0.99)	0.46 (0, 0.78)	0.88 (0,69 0.96)	0.83 (0.46, 0.94)	0.94 (0.87, 0.98)	0.84 (0.59, 0.94)	0.996 (0.987, 0.999)	0.94 (0.87, 0.98)	0.98 (0.96, 0.99)
70% MVIC	0.30 (0, 0.68)	0.97 (0.79, 0.99)	0.61 (0.15, 0.85)	0.96 (0.15, 0.85)	0.54 (0.10, 0.81)	0.98 (0.94, 0.99)	0.78 (0.47, 0.92)	0.95 (0.87, 0.98)	0.55 (0.10, 0.83)	0.996 (0.988, 0.999)	0.88 (0.72, 0.96)	0.98 (0.95, 0.99)
MVIC	0.02 (0, 0.53)	0.99 (0.96, 0.99)	0.65 (0.17, 0.87)	0.92 (0.82, 0.97)	0.66 (0.24, 0.87)	0.99 (0.98, 1.00)	0.78 (0.31, 0.93)	0.97 (0.92, 0.99)	0.69 (0.30, 0.88)	0.999 (0.998, 1.00)	0.97 (0.94, 0.99)	0.97 (0.91, 0.99)

MVIC, maximal voluntary isometric contractions.

**Figure 2:**
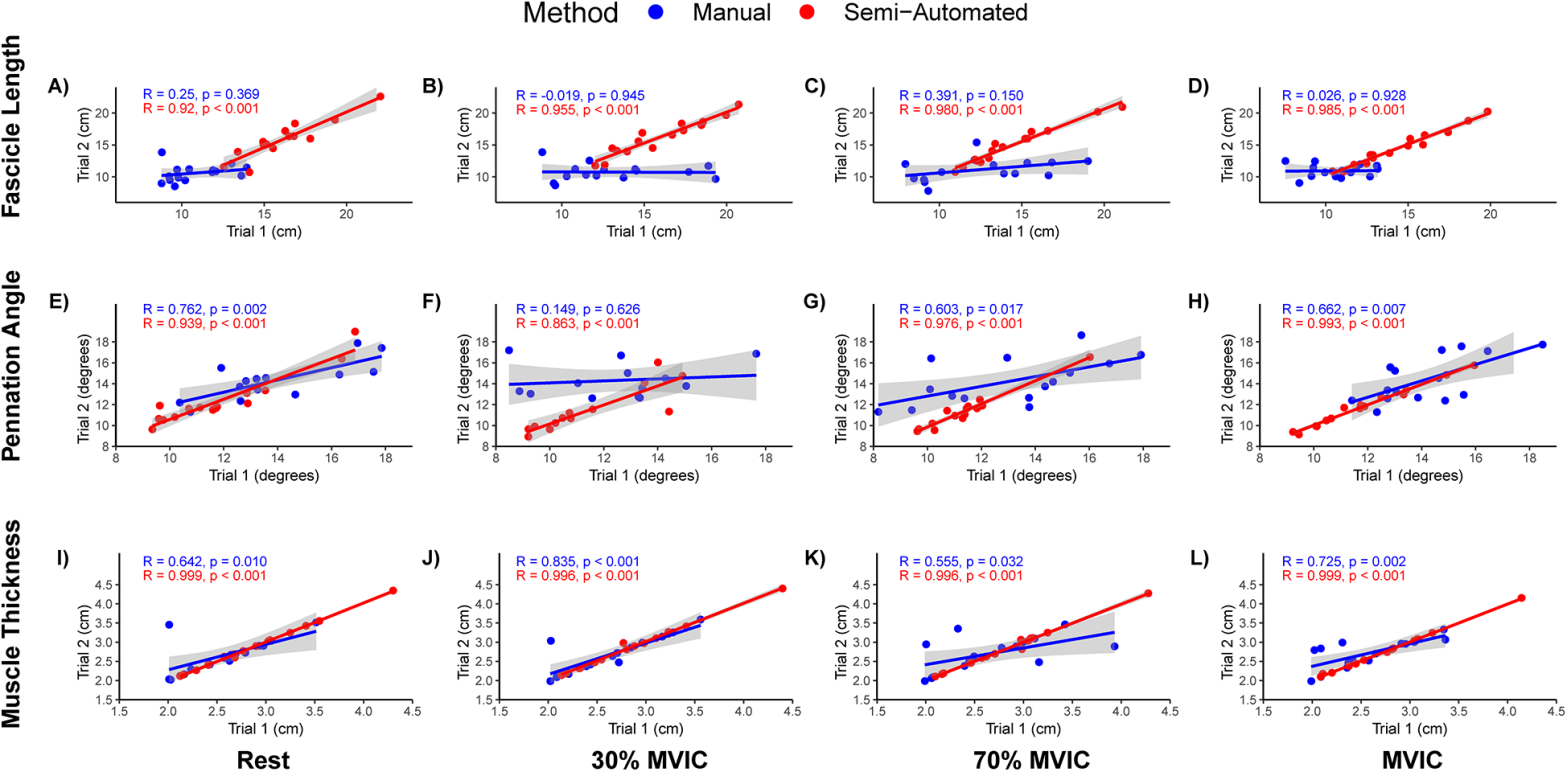
Scatterplots and Pearson's correlation of fascicle length, pennation angle, and muscle thickness intra-rater measures. **(A**–**D)** Fascicle lengths at rest, 30% maximal voluntary isometric contraction (MVIC), 50% MVIC, 70% MVIC, and MVIC, respectively. **(E**–**H)** Pennation angles at rest, 30% MVIC, 50% MVIC, 70% MVIC, and MVIC, respectively. **(I**–**L)** Muscle thickness at rest, 30% MVIC, 50% MVIC, 70% MVIC, and MVIC, respectively. A single rater for the manual and semi-automated methods analyzed the same images on two separate days (Trial 1 and Trial 2). Manual measures are presented in blue and semi-automated measures are shown in red.

The Pearson's correlation coefficients (*r*) across trial for measures from single rater are presented as *r* (95% confidence interval). Manual measurements between trials for the single novice rater for FL resulted in *r* = 0.25 (−0.30, 0.68), −0.02 (−0.52, 0.50), 0.39 (−0.15, 0.75), and 0.03 (−0.49, 0.53) for resting, 30% MVIC, 70% MVIC and MVIC intensities, respectively (Figures 2A–D). The between trials pennation angle *r* = 0.79 (0.45, 0.93), 0.59 (0.11, 0.84), 0.60 (0.13, 0.85), and 0.66 (0.23, 0.88) for resting, 30% MVIC, 70% MVIC and MVIC intensities, respectively (Figures 2E–H). Muscle thickness correlation between trials was *r* = 0.64 (0.19, 0.87), 0.83 (0.56, 0.94), 0.55 (0.06, 0.83), and 0.72 (0.34, 0.90) for resting, 30% MVIC, 70% MVIC and MVIC intensities, respectively (Figures 2I–L).

**Figure 3:**
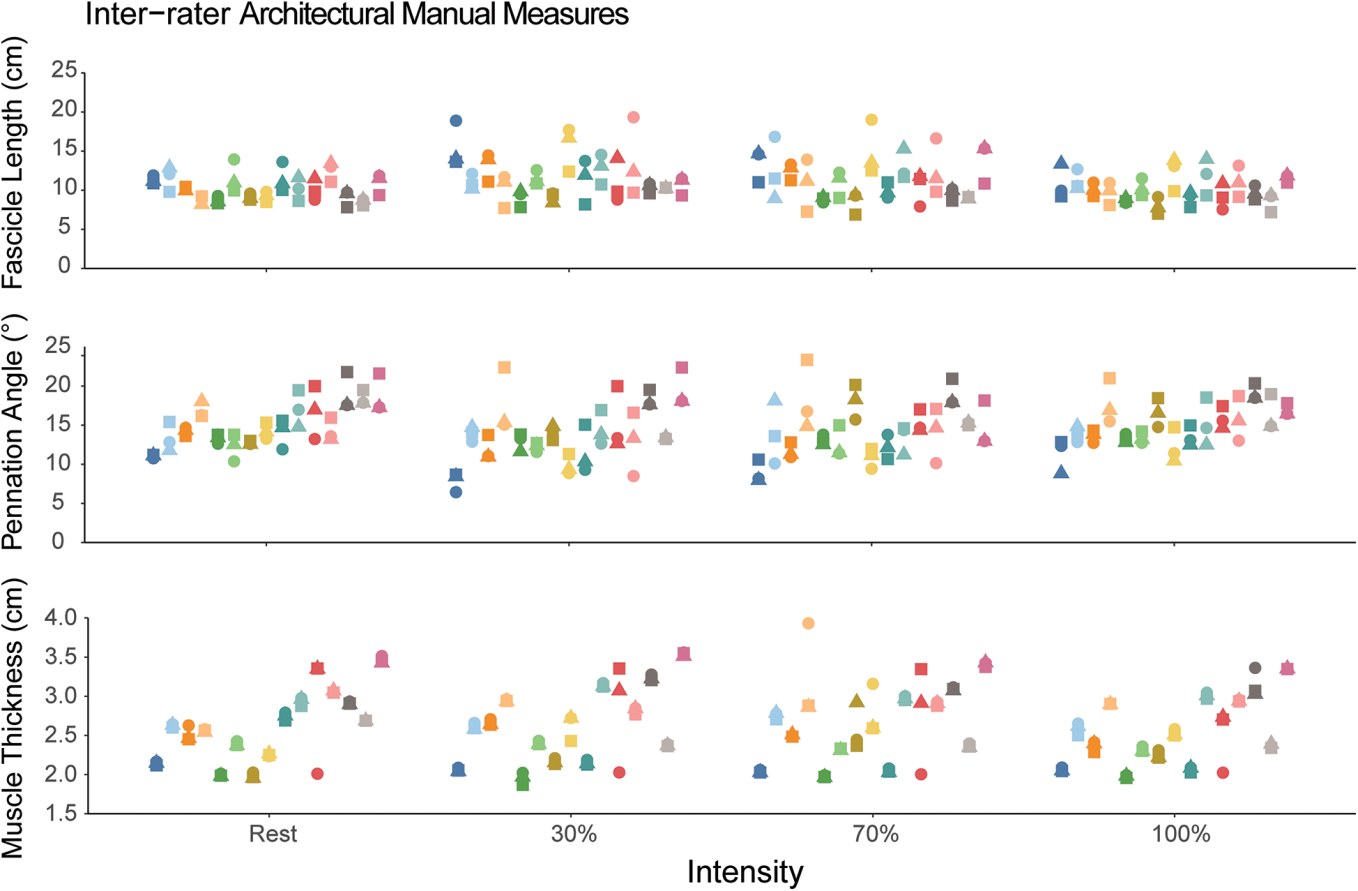
Manual measures of fascicle length, pennation angle, and muscle thickness. Each participant is represented as a different color and within a single column across each intensity. The different shapes correspond to the different novice raters. Each individual data point corresponds to the value measured by the rater. The dispersion of the data points within each column demonstrates the measurement inconsistency between the three raters.

The inter-rater ICC values for manual measures of FL, PA and muscle thickness for each contraction intensity are also shown in Table 1. The inter-rater reliability for manual measures of FL was moderate with ICC(2,k) values ranging from 0.61–0.75. The inter-rater reliability for pennation angle was good with ICC(2,k) values ranging from 0.78–0.84. The inter-rater reliability for muscle thickness was good to excellent with ICC(2,k) values ranging from 0.88–0.97. The measures for each rater are also shown visually in Figure 3.

### Semi-Automated measures

**Figure 4:**
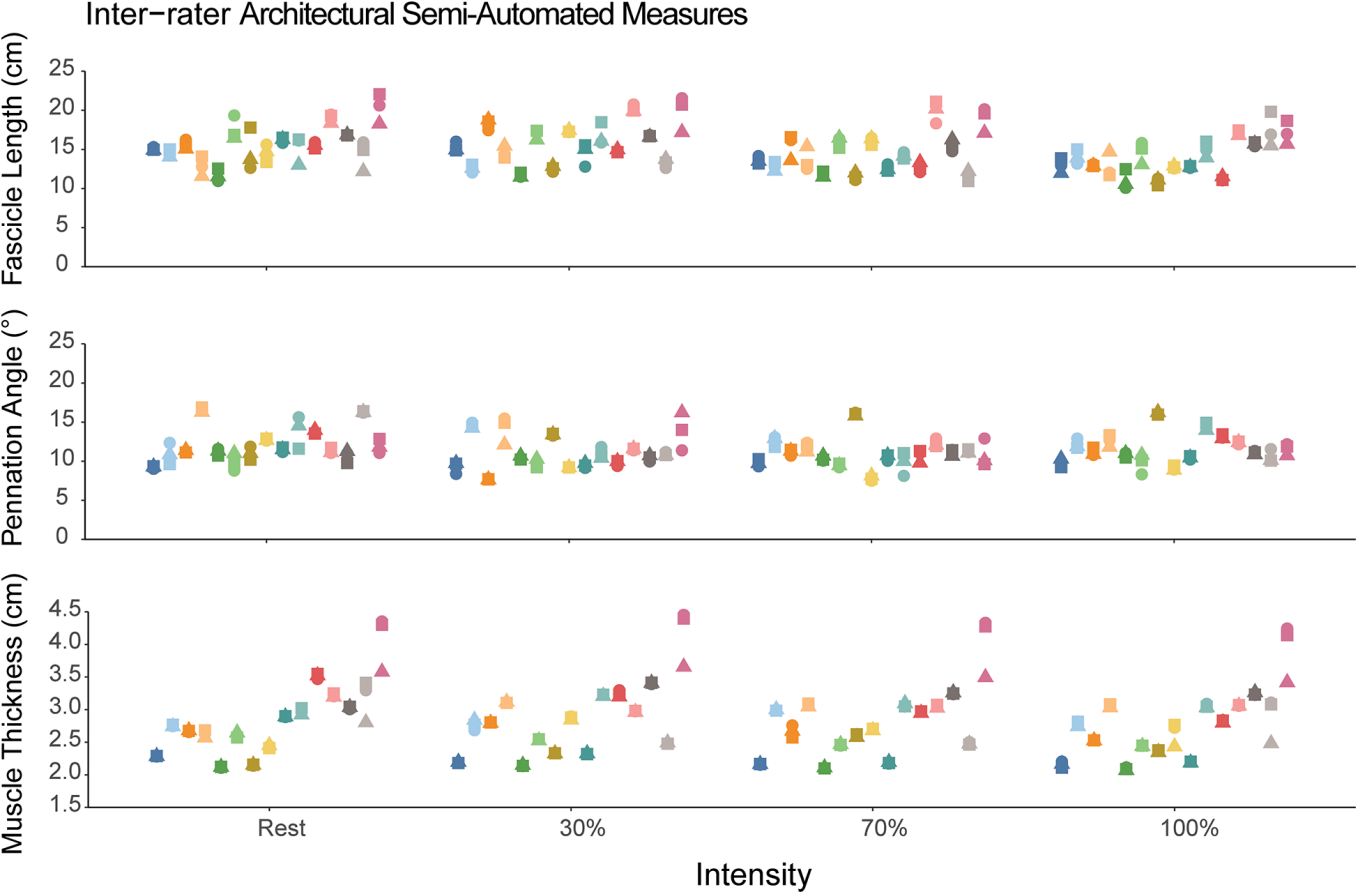
Semi-automated measures of fascicle length, pennation angle, and muscle thickness. Each participant is represented as a different color and within a single column across each intensity. The different shapes correspond to the different raters using the semi-automated method. Each individual data point corresponds to the value measured by the semi-automated algorithm. The dispersion of the data points within each column demonstrates the measurement consistency between the three raters.

The mean total run time for the semi-automated algorithm was approximately 88 s [median (interquartile range) = 65 (56–76) seconds, range: 45–366 s] with most of the time spent in the user isolating the aponeuroses. The intra-rater reliability for the semi-automated measures are shown in Table 1. The intra-rater reliability was good to excellent for FL (range: 0.90–0.99), PA (range: 0.88–0.99), and muscle thickness (range: 0.996–0.999) across all contraction intensities. The inter-rater reliability for semi-automated measures of FL was good to excellent [ICC(2,k) range: 0.89–0.96], while reliability was excellent for PA [ICC(2,k) range: 0.94–0.97] and muscle thickness [ICC(2,k) range: 0.97–0.98]. Figure 4 shows the semi-automated measures for each rater.

The Pearson's correlation coefficient between semi-automated measurement trials for the single novice rater for FL was *r* = 0.91 (0.76, 0.97), 0.95 (0.87, 0.99), 0.98 (0.94, 0.99), and 0.98 (0.95, 0.99) for resting, 30% MVIC, 70% MVIC and MVIC intensities, respectively (Figures 2A–D). The correlation coefficient between trials for pennation angle was *r* = 0.94 (0.82, 0.98), 0.88 (0.67, 0.96), 0.98 (0.94, 0.99), and 0.99 (0.98, 0.99) for resting, 30% MVIC, 70% MVIC and MVIC intensities, respectively (Figures 2E–H). The correlation between trials for muscle thickness was *r* = 0.999 (0.997, 0.999), 0.996 (0.987, 0.999), 0.996 (0.987, 0.999), and 0.999 (0.997, 0.999) for resting, 30% MVIC, 70% MVIC and MVIC intensities, respectively (Figures 2I–L).

### Comparison between manual and semi-automated measures

**Figure 5:**
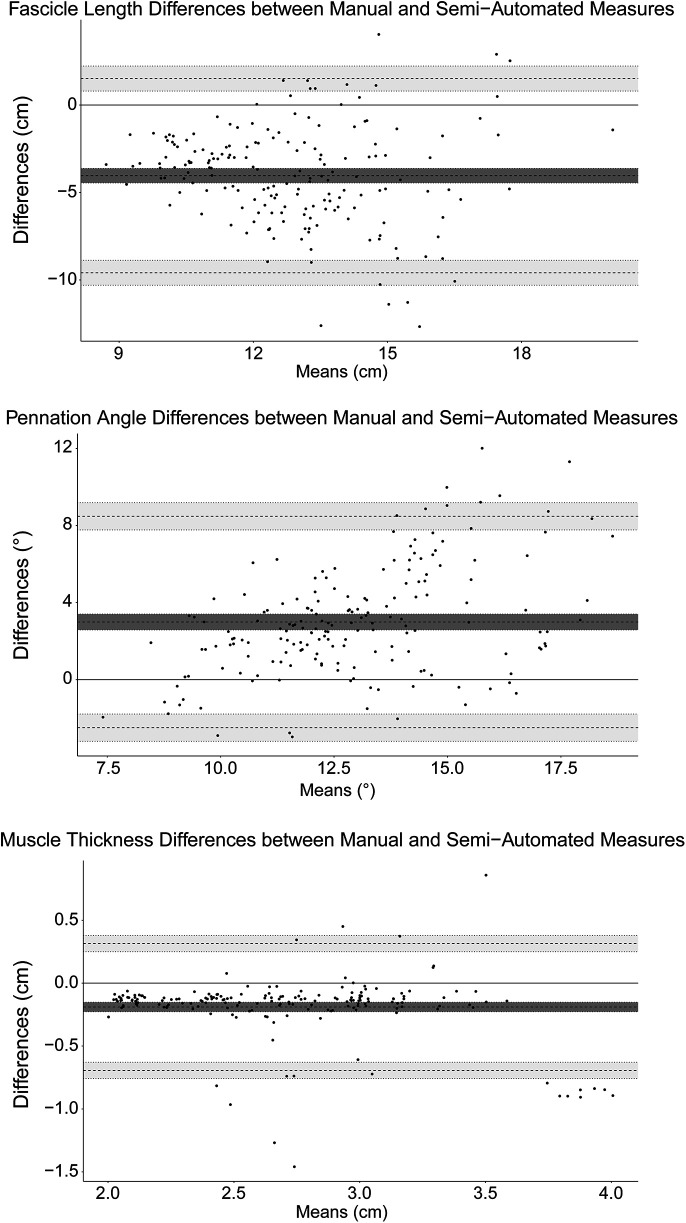
Bland-Altman plots for fascicle length, pennation angle, and muscle thickness. The mean bias is marked by the solid black line and the limits of agreement are marked by the dashed lines. The shaded regions correspond to the 95% confidence intervals for the bias and limits of agreement.

Measured values of FL, PA, and muscle thickness for manual and semi-automated measured are shown in Table 2. When comparing the difference between the manual and semi-automated measures, there was a bias of −4.0 cm (Limits of agreement, LoA: −9.59, 1.51) in FL, 2.99° (LoA: −2.49, 8.48) in PA, and −0.19 cm (LoA: −0.69, 0.31) in muscle thickness. The Bland-Altman plots for FL, PA, and muscle thickness are shown in Figure 5.

**Table 2:** Manual and semi-automated measures of fascicle length, pennation angle, and muscle thickness from three novice raters. Data are presented as mean (standard deviation).

Contraction Intensity	Fascicle length (cm)		Pennation angle (°)		Muscle thickness (cm)	
	Manual	Semi-automated	Manual	Semi-automated	Manual	Semi-automated
Rest	10.2 (1.6)	15.6 (2.4)	15.1 (2.8)	12.1 (2.2)	2.6 (0.4)	2.8 (0.5)
30% MVIC	11.7 (2.7)	15.7 (2.7)	13.7 (2.6)	11.0 (2.1)	2.6 (0.5)	2.8 (0.5)
70% MVIC	11.4 (2.7)	14.4 (2.6)	14.1 (3.4)	11.0 (1.9)	2.7 (0.5)	2. 8 (0.5)
MVIC	10.1 (1.8)	13.9 (2.3)	15.0 (2.6)	11.7 (1.9)	2. 6 (0.4)	2.8 (0.5)

MVIC, maximal voluntary isometric contractions.

## Discussion

This study investigated the reliability of vastus lateralis architecture measured by novice raters using both manual measurement procedures and a semi-automated algorithm across different contraction intensities. Intra-rater reliability for manual measures was poor to good, whereas the intra-rater reliability for semi-automated algorithm was good to excellent. Inter-rater reliability for architecture was moderate to good for manual measures and good to excellent for semi-automated measures. There was a bias in both FL and PA between the two measurement methods, but minimal differences were observed in muscle thickness measured between manual and semi-automated methods.

The intra-rater reliabilities of FL and PA were poor to good (ICC range: 0–0.77). Additionally, the FL and PA reliability decreased slightly with increasing contraction intensity, though muscle thickness intra-rater ICC appeared to be independent of contraction intensity (Table 1). Two reviews have indicated that FL and PA reliability is high (21, 33), even without formalized training in ultrasound imaging (21). Specifically, of the nine original investigations that analyzed architectural features of the vastus lateralis using traditional sonography, five studies did not mention the level of training for those who performed the analysis (40–44), while the others only mentioned that a single examiner performed the analysis (45–48). Our results differ from these reviews and show the intra-rater reliability for FL and PA was much lower than what had been reported previously. We attribute this lower reliability to the novice experience level of the rater. Though the rater was given instructions on the procedures and had practiced extracting architectural measures from ∼20 images from a separate data set of vastus lateralis ultrasound images, the rater had not been trained on hundreds of images as has been reported elsewhere (29). The decision to use novice raters was made to simulate what may be encountered in research labs that use ultrasound to measure architectural features—that is, untrained individuals (<2 h of guided, supervised instruction with feedback) are given instructions and asked to measure these features (22). Additionally, there are several steps within the process of manually measuring fascicles that might introduce error (e.g., scaling the image, identifying fascicles and aponeuroses, extrapolating the linear projection of the fascicle beyond the image to the intersection with the aponeurosis). Our findings support the notion that though formalized training in ultrasound imaging may not be required to obtain reliable measures of FL and PA, a stringent training protocol that includes practicing on a large set of images should be implemented for individuals who may be extracting muscle architectural measures.

The use of semi-automated algorithms to extract muscle FL and PA from static images and videos has grown within the last several years. Many algorithms are open-source, which makes them an accessible research tool (25, 26, 49). Though the use of semi-automated algorithms is not novel, the algorithm developed and used in the current study differs slightly. The developed semi-automated algorithm is easy to use and does not necessitate pre-cropping the image prior to analysis as is observed in other semi-automated algorithm workflows. Additionally, the algorithm used here does not apply filtering methods, such as a Hough transform, or use wavelet analysis (26, 49). Filtering the image prior to analysis aids in isolating fascicles by eliminating smaller echoes but may not be directly comparable to manual selection of fascicles. We did not include filtering techniques within our algorithm so that measurement comparisons would be more similar across manual analysis techniques. This approach has also been used previously with high intra- and between-day reliability (23).

In contrast to the manual measures, the intra-rater reliability for FL, PA and MT was all good to excellent (ICC range: 0.88–0.999) using the semi-automated algorithm. These values are consistent with other semi-automated methods of extracting FL, PA, and muscle thickness from ultrasound images in lower extremity muscles (23, 24, 27, 50). Additionally, reliability was not influenced by contraction intensity, suggesting these methods are robust to changes in muscle shape during contraction (33–36). Though we did not specifically calculate run time in the current investigation, a separate study measured architectural features of the biceps femoris long head (BFlh) muscle (51). Though the manual measurement procedures included additional measures to calculate fascicle lengths according to manual extrapolation methods and equations (29), the average time to extract 3 measures of FL, PA, and MT at the mid-belly of the BFlh muscle and input the corresponding data into a spreadsheet was approximately 10 min per image (unpublished data). Coupled with the speed of the analysis (less than 90 s per image) and the high intra-rater reliability when using the semi-automated approach, our findings suggest that muscle architecture measures can be quickly and reliably extracted from ultrasound images by novice raters.

The inter-rater reliability was moderate for FL whereas PA and MT reliability was good to excellent in the manual measures. In contrast, the semi-automated method had good to excellent reliability across FL, PA, and muscle thickness measures. Inter-rater reliability across three novice raters was much higher than intra-rater reliability, suggesting that averaging FL, PA and muscle thickness acquired across multiple novice raters may provide greater confidence in architectural measures. The inter-rater reliability for the semi-automated algorithm was similar to the intra-rater reliability and may suggest that a single rater may be able to achieve reliable results in an efficient method when considering the substantial time required for multiple raters to separately analyze each individual image within a set.

When assessing differences between the two methods, we observed FL 4 cm longer, PA 3° less, and comparable muscle thickness with the semi-automated algorithm compared to manual measures. Despite the observed differences in FL and PA measures between manual and semi-automated approaches, both methods resulted in values consistent with those previously reported. In healthy adults, FL and PA have been reported to range from 8–14 cm and 10–20°, respectively, across resting and contracted conditions, different knee joint angles, and using both traditional B-mode images and linear extrapolation methods and extended field-of-view (EFOV) imaging (9, 10, 16, 23, 31, 42, 52–54). Ando et al. observed FL and PA in the vastus lateralis measured at 12.2, 11.7, and 11.0 cm and 13.2, 11.3, and 12.8° when at rest, 30% and 50% MVIC, respectively (53). However, the authors used EFOV imaging to acquire images, which measures shorter FL (∼4%–9%) and smaller PA (∼1%–9%) when compared to linear extrapolation methods used in the current study (28, 29). Due to the observed variability in FL and PA measured by our novice raters, we cannot directly state which method is more valid. Future developments of the algorithm to determine its validity to measures acquired from expert raters or comparisons between other semi-automated procedures are needed. For example, one such improvement might include only fascicles where the linear path of the fascicle inserting into the superficial and deep aponeuroses has no more than 20% of the calculated length extending beyond the borders of the image to minimize the length estimated beyond the image (28). Future lines of research include studies investigating the validity of measures derived from the semi-automated method in different muscle groups (e.g., hamstrings, gastrocnemius muscles), in comparison to different equations of linear extrapolation (29), and how regional variation in muscle architecture may influence the reliability of semi-automated measures (55, 56).

Though we showed higher reliability with semi-automated measures of FL and PA compared to manual analyses, the study did have limitations. The sample size was small with only *N* = 15 and was limited to healthy individuals who met certain physical activity and strength levels. It is unclear if the reliability of both methods would change in populations with disease, pathophysiology, or injury. By design, the raters were novice raters which likely influenced the lower reliability in manual measures. However, this decision to only include novice raters prevented optimization of the algorithm to “true” measures of FL, PA, and muscle thickness. The semi-automated algorithm should also be tested and validated against measures from trained raters and in different pennate muscles to determine its generalizability.

## Conclusion

The findings of this study suggest that the reliability of vastus lateralis architecture measured by novice raters is lower when measuring FL and PA manually compared to semi-automated algorithms. Architectural measure reliability using manual measures might be increased by implementing a stringent training protocol with a large set of practice images for individuals analyzing ultrasound images and by averaging FL, PA and muscle thickness acquired across multiple novice raters. In contrast, muscle architecture measures can be quickly and reliably extracted from ultrasound images by novice raters using semi-automated algorithms. Despite the ease and efficiency of these algorithms, they should still be validated against manual measures extracted from experienced raters.

## Data Availability

The datasets generated and/or analyzed during the current study are available from the corresponding author on reasonable request.
